# On Medical Reform

**Published:** 1810-03

**Authors:** 


### *To the Editors of the Medical and Physical Journal.*

GENTLEMEN,

AMONG the various papers which have been published in your Journal, on the existing state of the Medical Profession in this Empire, I do not recollect to have noticed one from Essex. This induces me to offer you what appears to me to be the most prominent features of our situation.

To begin with our M. D.'s, they are unquestionably men of learning and professional skill, and many of them act from honourable motives for the interest and welfare of the inferior branches of the profession; I wish I could say thus much for "all." But if a physician who enjoys a very extensive and lucrative practice, descends beneath the dignity of his profession, in recommending (either in a direct or indirect manner) his prescriptions to a favourite druggist, to the material injury of the apothecary, whom he studiously avoids; and when compelled to meet him, conducts himself with all the parade of conscious superiority and indifference, far unlike that confidence and respect which ought to be kept up between the physician and apothecary, not only for their individual comfort, but for the safety of the patient: I say, if a physician conducts himself thus, he can only be biased by selfish and unworthy motives, disgraceful to his character and profession; but disgraceful as it is, truth bids me declare, that one instance at least of this kind exists among us.—I am disposed to believe that most of the retail druggists are able to compound a prescription faithfully, but still it is not their province, and the general practice of it lessens materially the honest and necessary emoluments of the apothecary. I say "necessary," for surely an apothecary ought to obtain a respectable and comfortable competence for his labour.—We have also a physician who prescribes and compounds his own medicines, and also practices as a surgeon and accoucheur.

coucheur. This I apprehend is not a very unusual case of irregularity.

Retail druggists have very much increased within a short period of time. This appears to arise from the custom of their compounding prescriptions becoming daily more prevalent, and from this custom we may fairly date the origin of a very serious event confined to the lower classes of the community. For by compounding prescriptions, the druggist acquires the knack of "prescribing" himself! thus uniting in his own person, the offices both of "physician and apothecary." Many of them acquire by this means no inconsiderable practice among the poor, their charges being lower than those of the apothecary; at present, this it appears can only be prevented by regular practitioners adapting their charges to the abilities of the patient; perhaps this is now practised to a certain extent, though not sufficiently. A man actuated by benevolent motives of this kind, will eventually obtain more deserved reputation, respect, and even emolument, than he who insists upon the same remuneration from all patients indiscriminately. I wish this spirit of liberality was more prevalent; where it does reside, the druggist can do but little harm.*

Uneducated men, stiling themselves "surgeon-apothecaries" and men-midwifes, are with us not so numerous as in many parts of the kingdom; still we have a few whose ignorance and presumption are alike notorious. I know one of these shameless pretenders, who in attempting to divide the frenum of a child's tongue, thrust his scissars far enough to divide a branch of one of the lingual arteries, which brought on a hæmorrhage that destroyed the infant in forty-eight hours! The mother of this infant entertained a vulgar, and for the most part, an erroneous notion, that the child was tongue-tyed, and first brought it to a surgeon with whom I then resided. Upon examination, the frenum appeared naturally and perfectly conformed, but the foolish parent it seems could not be made to believe this.—On another occasion, this man pronounced the presenting part of a "Psoas abscess," a "Bubo!" and immediately set about salivating the sufferer, who was a married

* What is here said, does not apply to the poorest classes, because they are (what is usually termed) "farmed" for a stated salary. It is the inferior tradesmen I allude to; they are not allowed a "parish doctor," of course they pay for medical attendance themselves, though many of them can no better afford it than the husbandman.

married man. The poor fellow was convinced of his own innocence, and had no reason to suspect his wife's fidelity, and not being satisfied with this decision, he some time afterwards applied to several other surgeons, who immediately discovered the error, but too late to afford the man a hope of recovery, as the favourable moment had gone by, and the man's constitution was under the influence of mercury.*

Itinerant quacks are gradually disappearing, but we have many resident "lady doctors," who bountifully dispense "infallible remedies" for St. Anthony's fire, jaundice, sore breasts, &c. Their practice and fame surely ought to be confined to the lower classes, and yet it is no uncommon thing for opulent members of society, (who have little else to do, but to nurse their imaginary complaints,) to resort to this kind of quackery after having exhausted the patience and "skill" of their regular medical attendants.

I think the worst evil of all, as it effects the welfare of society, is the increased consumption of "patent medicines," connected with "domestic quackery." Buchan is the "oracle" which these domestic followers of Esculapius consult; with this book as a guide, parents do not hesitate to administer medicines to their children, servants, and dependants; perhaps nine times in ten no harm may result, but fatal diseases often make their approach in an insidious manner, and medical advice is only called in time enough to witness the last, but ineffectual efforts of nature; but not time enough to catch the favourable moment when the rapid strides of disease might be arrested, and the sufferer snatched from his impending fate.

Credulity and confidence are the constant companions of ignorance; and the mind is readily captivated by promises, especially if they are couched in ambiguous language. Empirics take advantage of these circumstances, to deceive a credulous public, and find a ready sale for their nostrums.

* I could adduce a parallel instance of ignorance in a youth who left the Borough Hospitals, about twelve months since, after having had all the advantages that the present system of education for a surgeon-apothecary affords. This "self-taught" genius applied a "hernial truss" to a "suppurating bubo!" This is a strong proof that the present mode of education adopted at the public schools in the metropolis, comprising a "general attendance on lectures," is too lax and insecure. The acquirements of the pupil should be ascertained by strict and impartial examinations, that the profession may be secured against the disgrace entailed upon it by the admission of unworthy members.

nostrums. They also receive the sanction of the legislature; and one would think they find but little difficulty in obtaining "noble" and "dignified" attestations to "surprising cures." That men of the first rank and preferment in society, should condescend to prostitute their names, to answer the sordid views of individuals, to the injury of the community, is a matter of astonishment as well as regret to your humble servant,

AN ESSEX PRACTITIONER.

*February* 7, 1810.

P. S. From the reputable testimonies adduced in your Journal respecting the medicinal powers of cobweb, I was determined to give it further trial, when opportunity offered; a few days since an inveterate ague presented itself. The patient had laboured under an irregular intermittent for many months, which at last assumed a regular quartan form. I prepared these pills, each containing five grains of the most recent webs that I could find, after a careful search. I directed the patient to take one of these pills at intervals of two hours, managing so that the last should be taken about half an hour before the expected fit. The man obeyed my directions punctually, but unhappily for him obtained no relief or abatement in his disorder, and the only effect he could attribute to the pills, was a pain in his bowels.

Since writing the above, I received a letter from a friend at Edinburgh, in which he informs me, that Dr. Gregory and the College of Physicians are at variance from an extraordinary cause. The Members of the College wish to make up their own prescriptions! This Dr. Gregory refused to accede to with scorn, and he is about to publish his reasons, or defence. I hope we shall soon hear more of this subject.

